# Supplementary material for: CYP154C5 Regioselectivity in Steroid Hydroxylation Explored by Substrate Modifications and Protein Engineering
Source: Chembiochem. 2020 Nov 30;22(6):1099–110. doi: 10.1002/cbic.202000735 (PMC8048783; doi:10.1002/cbic.202000735)
Supplement: Supplementary file 1 — Supplementary [file CBIC-22-1099-s001.pdf]

ChemBioChem

Supporting Information

CYP154C5 Regioselectivity in Steroid Hydroxylation Explored by Substrate Modifications and Protein Engineering**

Paula Bracco, Hein J. Wijma, Bastian Nicolai, Jhon Alexander Rodriguez Buitrago, Thomas Klünemann, Agustina Vila, Patrick Schrepfer, Wulf Blankenfeldt, Dick B. Janssen, and Anett Schallmey*

Table of Contents

1. Mutagenesis **2**

2. Protein purification **2**

3. Biochemical characterization of CYP154C5 mutants **3**

4. CYP154C5 modeling and docking **8**

5. Protein crystallography **13**

6. Product identification **14**

1. Mutagenesis

Table S1. Applied QuikChange® primers for the generation of CYP154C5 mutants. The introduced alanine codon is underlined.

Primer	Sequence (5’ to 3’)
M84A fwd	GCCGCTGATCGGCGCGATCGACGTGGAC
M84A rev	GTCCACGTCGATCGCGCCGATCAGCGGC
F92A fwd	GGACCGCTCGATGGCCACCGTGGACGGC
F92A rev	GCCGTCCACGGTGGCCATCGAGCGGTCC
Q239A fwd	TGATCGGCAATCTCGCGGCGCTCGTCGCC
Q239A rev	GCGACGAGCGCCGCGAGATTGCCGATCAG
Q398A fwd	CCCGTCCTCACCGCGAACGACCTGTCCCAC
Q398A rev	GTGGGACAGGTCGTTCGCGGTGAGGACGGG

2. Protein purification

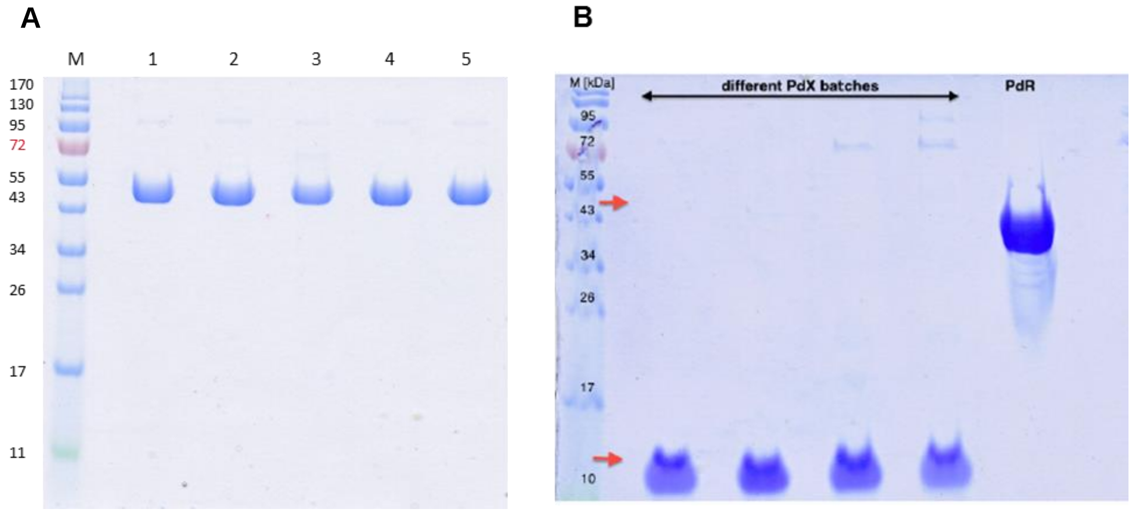

Figure S1. SDS-PAGE of purified proteins. **M** represents the Marker; Proteins shown correspond to **A**) 1: CYP154C5 (45.3 kDa); 2: CYP154C5 M84A; 3: CYP154C5 Q398A; 4: CYP154C5 Q239A; 5: CYP154C5 F92A and **B**) four different Pdx (11.5 kDa) batches; PdR (45.5 kDa).

3. Biochemical characterization of CYP154C5 mutants

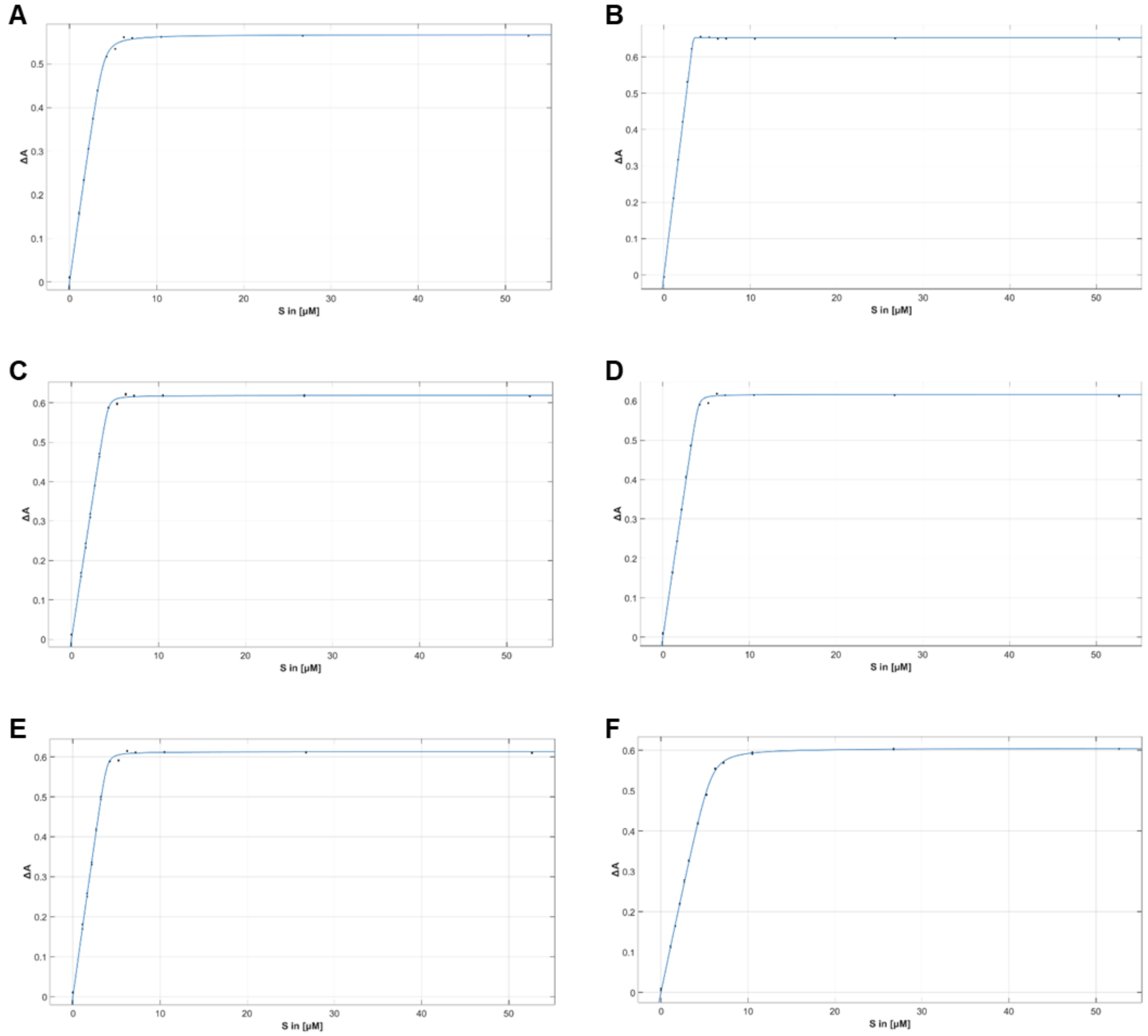

Figure S2. Substrate-binding titrations for CYP154C5 wild type using steroid substrates **A**: pregnenolone (**1**), **B**: dehydroepiandrosterone (**2**), **C**: progesterone (**3**), **D**: androstenedione (**4**), **E**: testosterone (**5**) and **F**: nandrolone (**6**). ΔA was plotted against the applied steroid concentration and the resulting data was fitted using the tight binding equation.

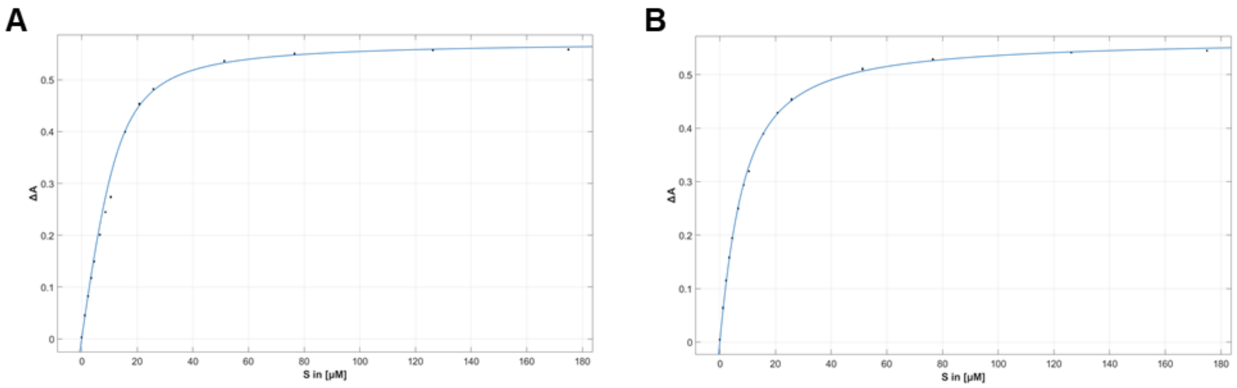

Figure S3. Substrate-binding titrations for CYP154C5 M84A using steroid substrates **A**: androstenedione (**4**) and **B**: testosterone (**5**). ΔA was plotted against the applied steroid concentration and the resulting data was fitted using the tight binding equation.

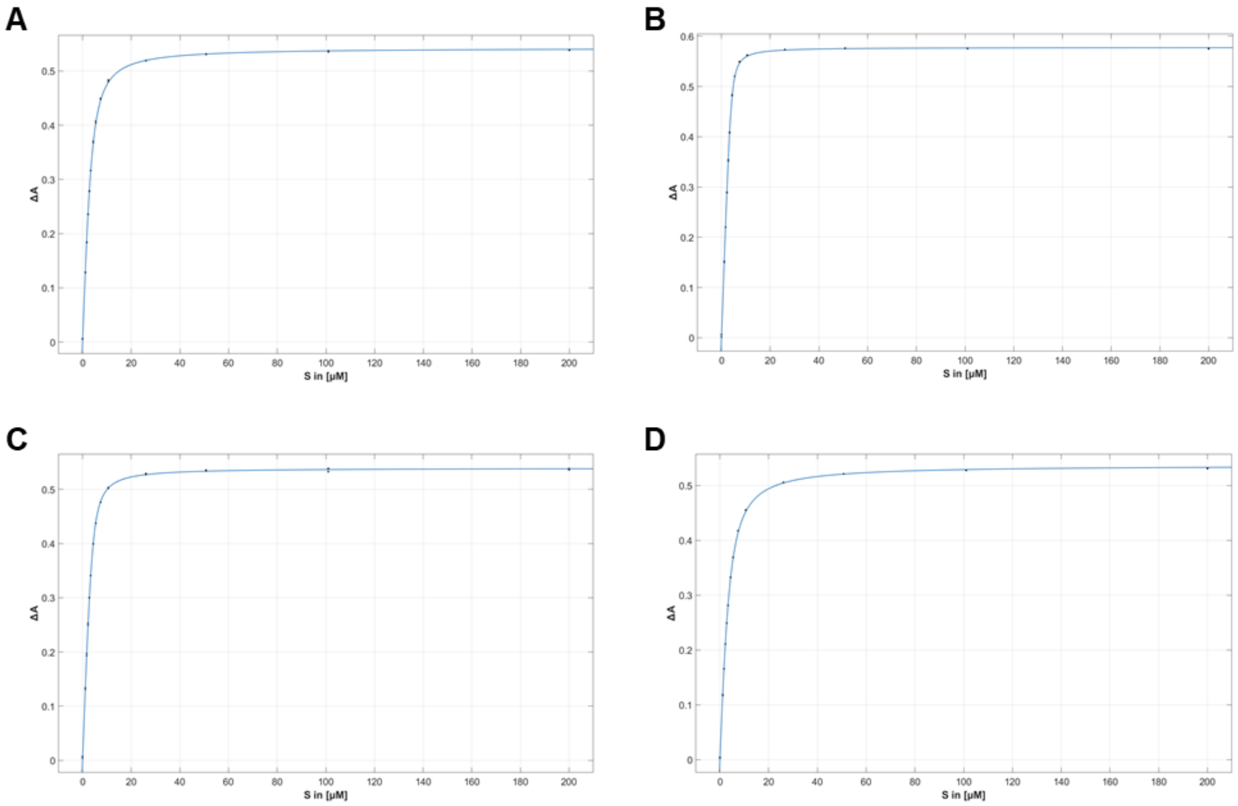

Figure S4. Substrate-binding titrations for CYP154C5 F92A using steroid substrates **A**: dehydroepiandrosterone (**2**), **B**: progesterone (**3**), **C**: androstenedione (**4**) and **D**: testosterone (**5**). ΔA was plotted against the applied steroid concentration and the resulting data was fitted using the tight binding equation.

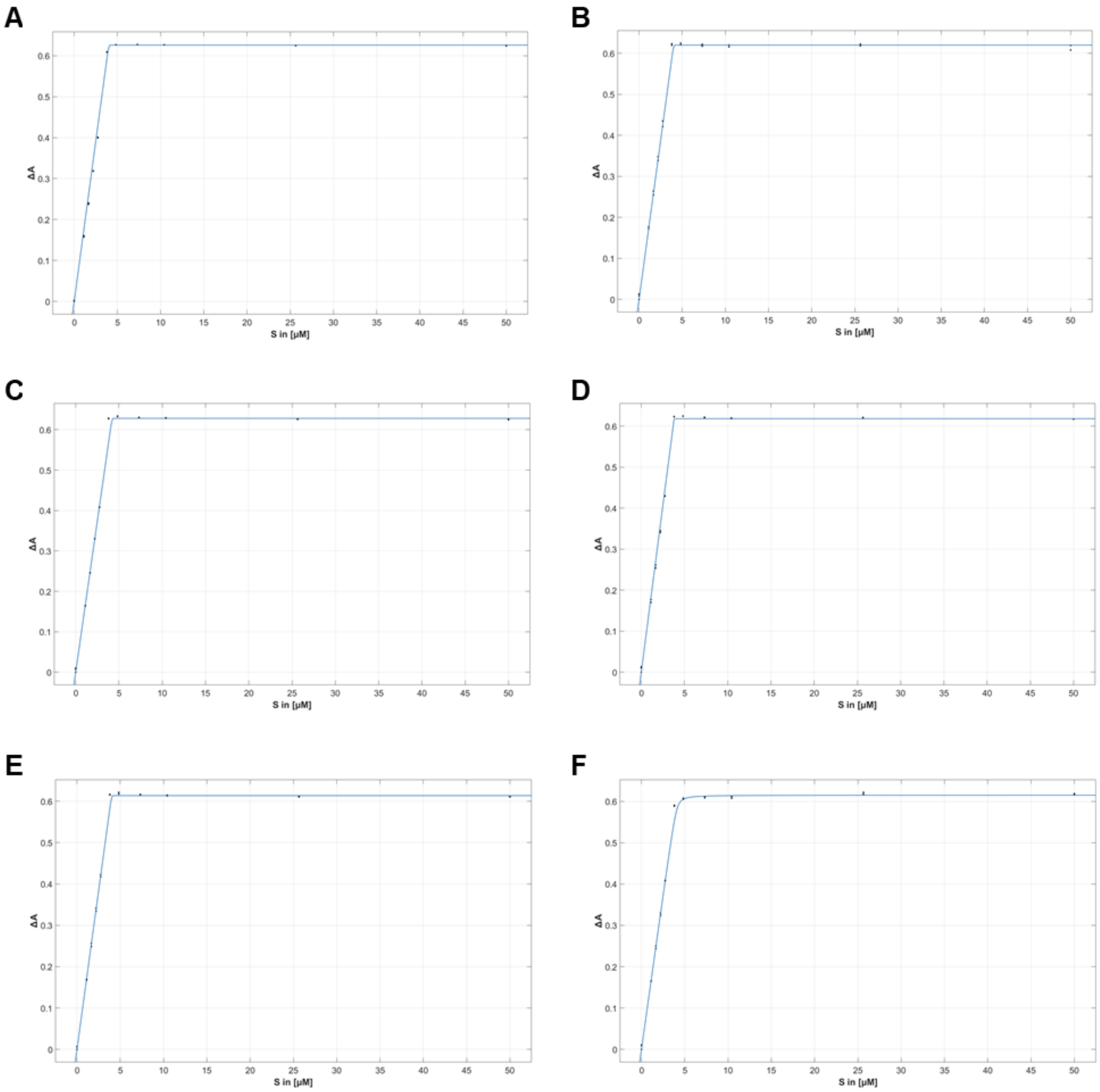

Figure S5. Substrate-binding titrations for CYP154C5 Q239A using steroid substrates **A**: pregnenolone (**1**), **B**: dehydroepiandrosterone (**2**), **C**: progesterone (**3**), **D**: androstenedione (**4**), **E**: testosterone (**5**) and **F**: nandrolone (**6**). ΔA was plotted against the applied steroid concentration and the resulting data was fitted using the tight binding equation.

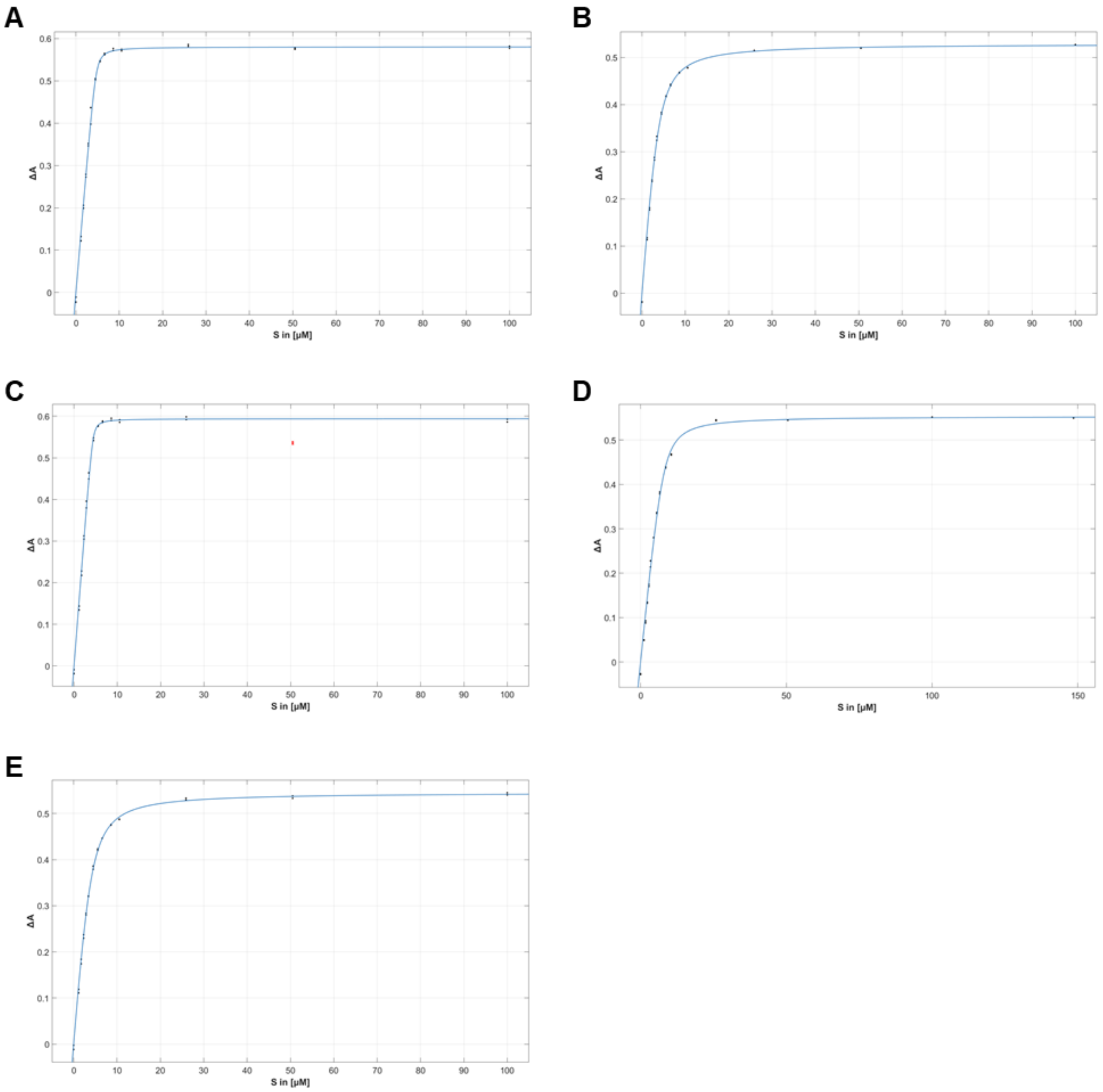

Figure S6. Substrate-binding titrations for CYP154C5 Q398A using steroid substrates **A**: pregnenolone (**1**), **B**: dehydroepiandrosterone (**2**), **C**: progesterone (**3**), **D**: androstenedione (**4**) and **E**: testosterone (**5**). ΔA was plotted against the applied steroid concentration and the resulting data was fitted using the tight binding equation. Red dots represent data points that were not included in the fitting.

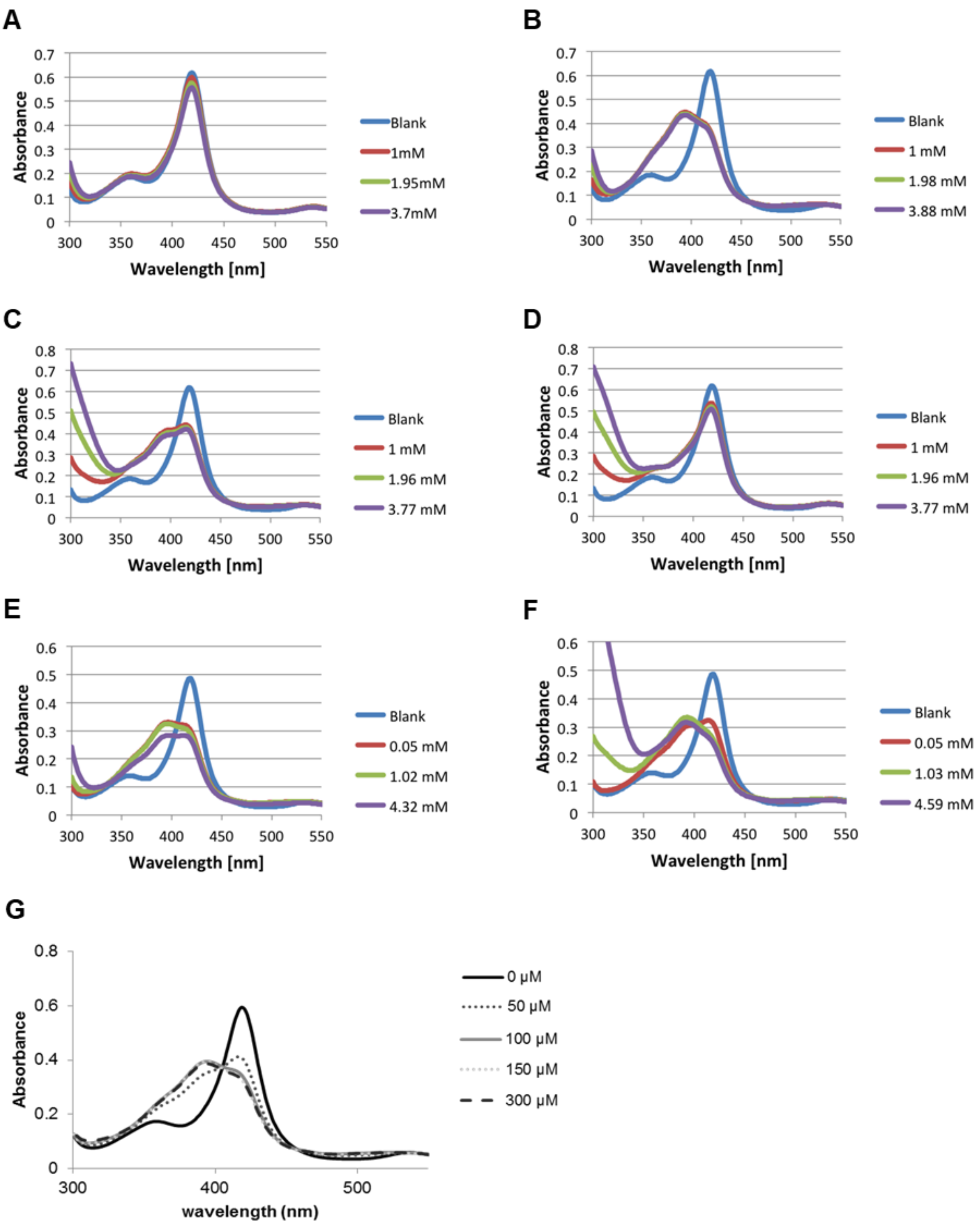

Figure S7. Substrate titrations for K_D determination. The following enzyme-substrate combinations resulted only in a partial or no spectral shift upon substrate addition: (**A**) CYP154C5 M84A with **1**; (**B**) CYP154C5 M84A with **2**; (**C**) CYP154C5 M84A with **3**; (**D**) CYP154C5 M84A with **6**; (**E**) CYP154C5 F92A with **1**; (**F**) CYP154C5 F92A with **6** and (**G**) CYP154C5 WT with **9**.

Table S2. Obtained conversion values for purified CYP154C5 wild type and mutants in the transformation of steroids **1-6** at 30°C after 8 h reaction time. Measurements were performed in duplicate.

Substrate	Conversion (%)				
	**CYP154C5**	**CYP154C5 M84A**	**CYP154C5 F92A**	**CYP154C5 Q239A**	**CYP154C5 Q398A**
Pregnenolone (**1**)	100 ± 0	8 ± 5	27 ± 1	99 ± 0	40 ± 2
Dehydroepi-androsterone (**2**)	100 ± 0	26 ± 1	83 ± 12	100 ± 0	39 ± 0
Progesterone (**3**)	100 ± 0	67 ± 1	83 ± 1	100 ± 0	82 ± 1
Androstene-dione (**4**)	100 ± 0	26 ± 0	59 ± 0	87 ± 1	100 ± 0
Testosterone (**5**)	74 ± 2	38 ± 1	58 ± 1	100 ± 0	28 ± 3
Nandrolone (**6**)	69 ± 3	–[a]	32 ± 0	100 ± 0	–[a]

[a] No conversion observed

4. CYP154C5 modeling and docking

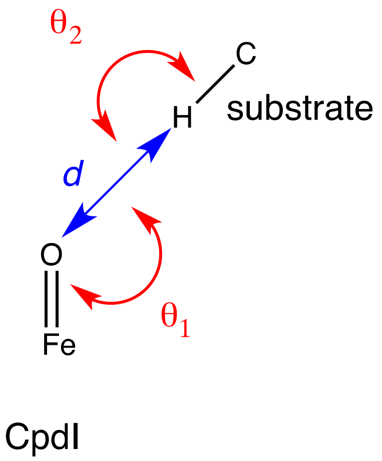

Figure S8. Geometric definitions of a near attack conformation for P450-catalyzed hydroxylation. Of the P450, only the iron and the reactive oxygen atom of compound I are shown while of the substrate only the attacked hydrogen and carbon atom are shown. A conformation was scored to be a NAC if it displayed simultaneously a distance $d \leq 2.72$ Å, an angle θ_1 of 100-140°, and an angle θ_2 of > 140°.

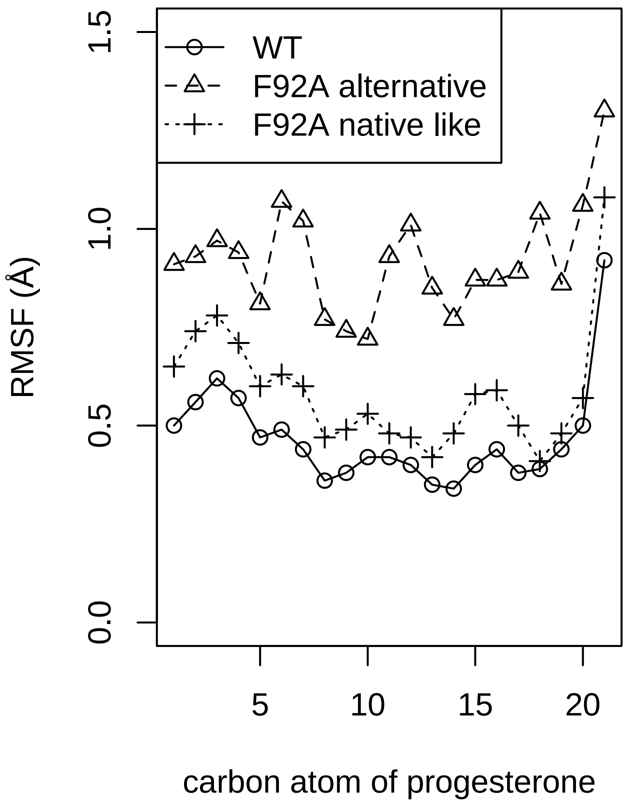

Figure S9. The positional flexibility of the substrate progesterone (**3**) increases due to the F92A mutation in CYP154C5. For each complex, the RMSF was calculated from three independent MD simulations of each 22 ns.

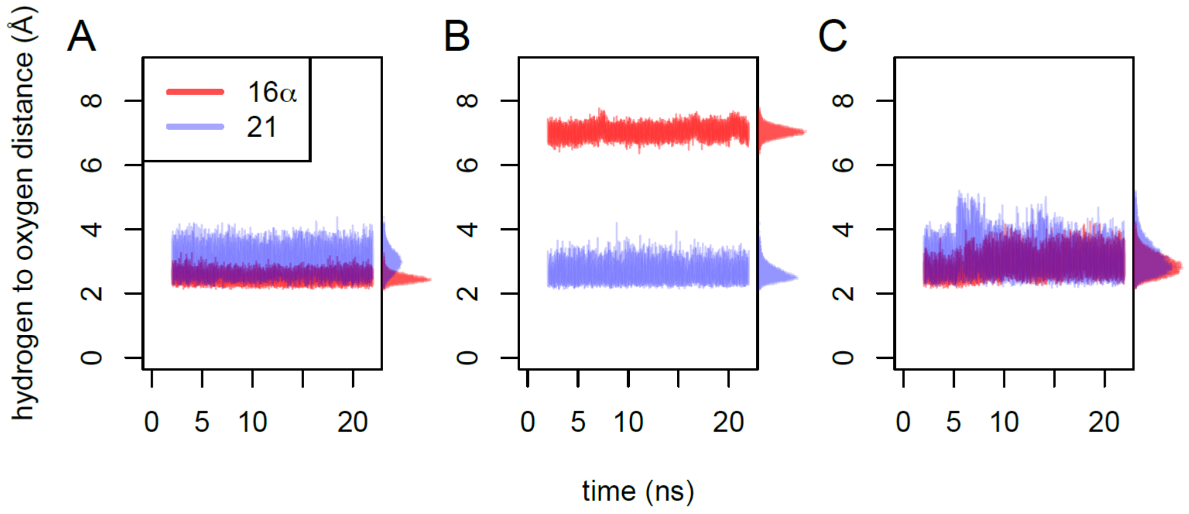

Figure S10. Distance of the substrate‘s (**3**) hydrogen atoms to the oxygen of compound I in trajectory 2. Only the shortest distance (there are three methyl hydrogens at position 21) is shown at each time-point. In this figure, the second out of three independent MD simulations is show (the first is shown in Figure 4 within the main manuscript, the third in Figure S11). **A**) Wild-type CYP154C5 with progesterone (**3**) bound. **B**) CYP154C5 F92A with progesterone (**3**) bound in the alternative orientation; **C**) CYP154C5 F92A with progesterone (**3**) bound in the native-like orientation.

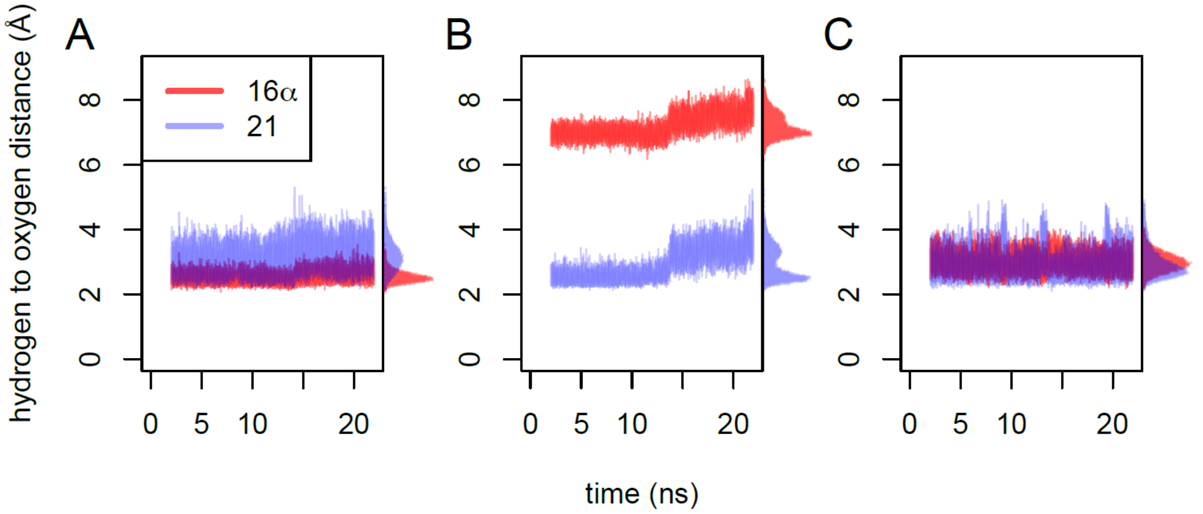

Figure S11. Distance of the substrate's (**3**) hydrogen atoms to the oxygen of compound I in trajectory 3. Only the shortest distance is shown at each time-point. In this figure, the third out of three independent MD simulations is show (the first is shown in Figure 4, the second in Figure S10). **A**) Wild-type CYP154C5 with progesterone (**3**) bound. **B**) CYP154C5 F92A with progesterone (**3**) bound in the alternative orientation; **C**) CYP154C5 F92A with progesterone (**3**) bound in the native-like orientation.

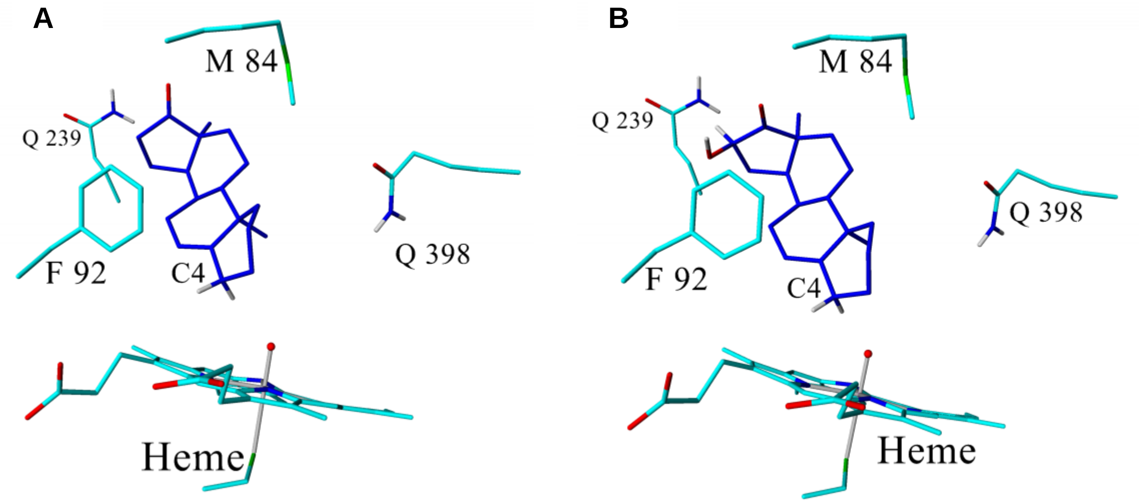

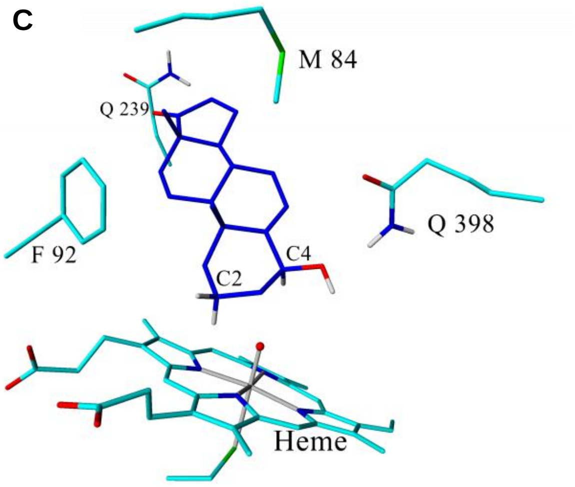

Figure S12. Obtained steroid orientations and potential hydroxylation sites during docking of **A**) 3-deoxydehydroepiandrosterone (**10**), **B**) 16α-hydroxy-3-deoxydehydroepiandrosterone and **C**) 4β-hydroxy-3-deoxydehydroepiandrosterone as substrates in CYP154C5 (as compound I model).

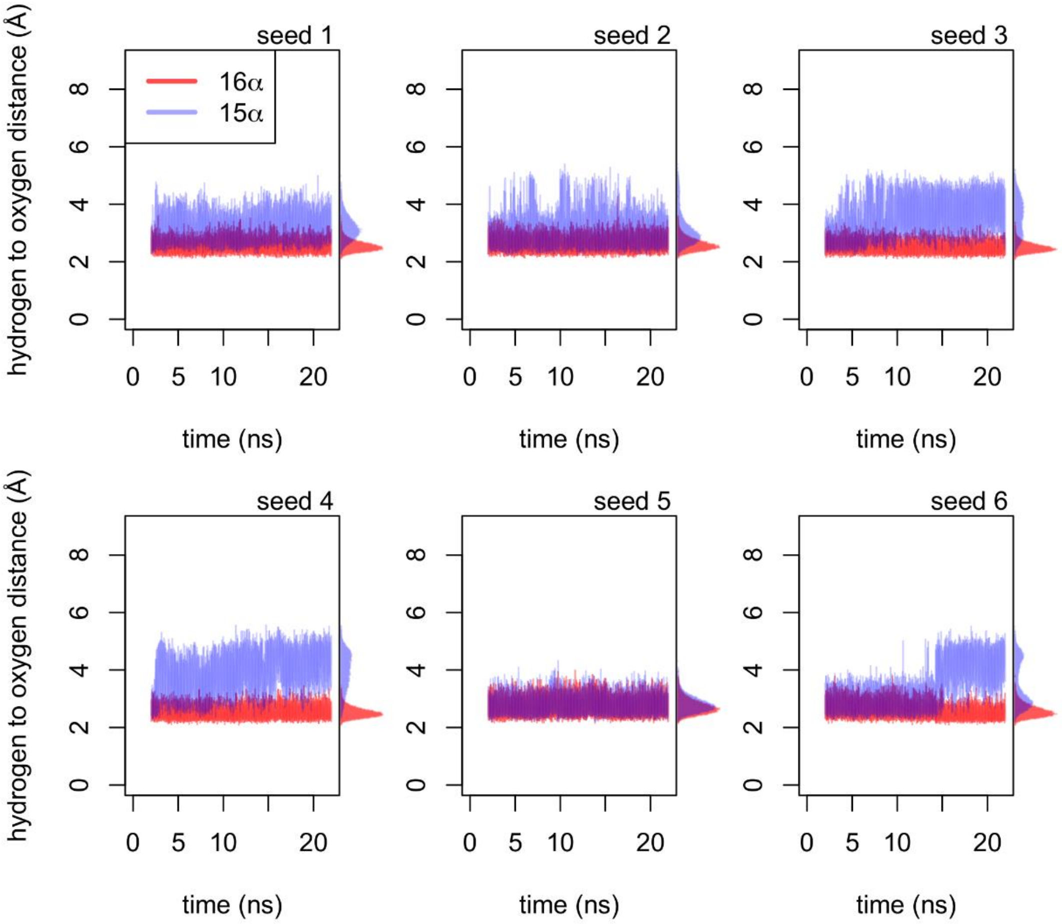

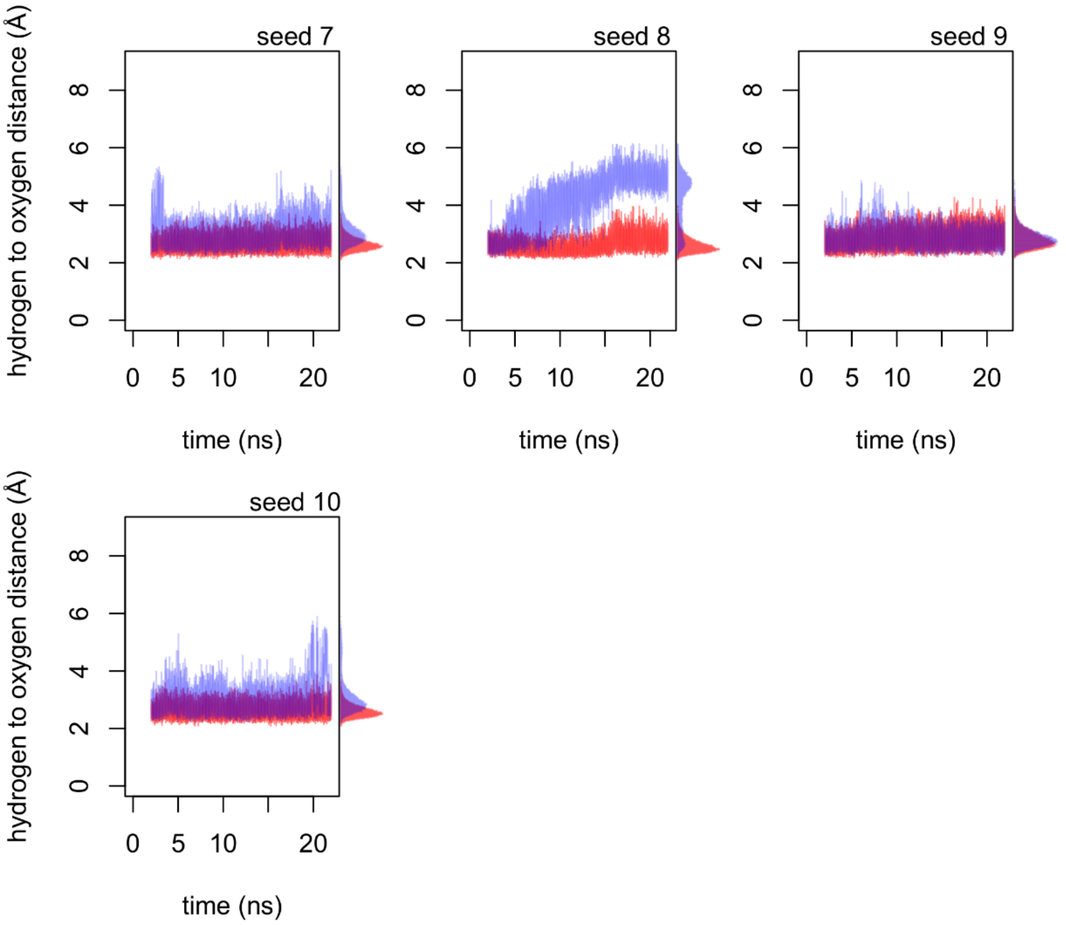

Figure S13. Distances of the substrate's (**11**) hydrogen atoms to the oxygen of compound I during MD simulation. Since significant differences were observed within the 10 individual MD trajectories, the results of all trajectories are shown individually.

5. Protein crystallography

Table S3. Data collection and refinement statistics for CYP154C5 co-crystallized with 5α-androstan-3-one (**11**). Values in parentheses are for the highest resolution shell.

**Data collection**	
Wavelength	1.000
Resolution range	46.6 - 2.0 (2.072 - 2.0)
Space group	R 3 H
Unit cell	103.411 103.411 218.21 90 90 120
Total reflections	618601 (58073)
Unique reflections	58736 (5859)
Multiplicity	10.5 (9.9)
Completeness (%)	99.84 (99.62)
Mean I/sigma(I)	17.11 (0.96)
Wilson B-factor	31.29
R-merge	0.3117 (1.848)
R-meas	0.3274 (1.949)
R-pim	0.09959 (0.614)
CC1/2	0.976 (0.496)
CC*	0.994 (0.814)
**Model refinement**	
Reflections used in refinement	58678 (5842)
Reflections used for R-free	2989 (303)
R-work	0.2073 (0.2963)
R-free	0.2479 (0.3520)
CC(work)	0.924 (0.716)
CC(free)	0.885 (0.623)
Number of non-hydrogen atoms	6917
macromolecules	6286
ligands	128
solvent	503
Protein residues	810
RMS(bonds)	0.009
RMS(angles)	0.85
Ramachandran favored (%)	97.63
Ramachandran allowed (%)	2.73
Ramachandran outliers (%)	0.00
Rotamer outliers (%)	0.30
Clashscore	2.19
Average B-factor	38.27
macromolecules	38.35
ligands	28.54
solvent	39.76
Number of TLS groups	15

6. Product identification

a. Conversion of progesterone (3) by CYP154C5 F92A

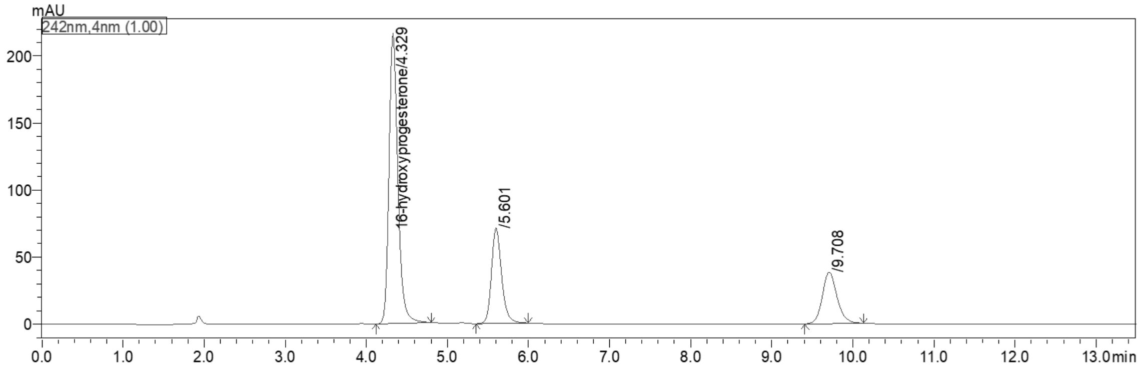

Figure S14. HPLC chromatogram of progesterone (**3**) conversion after 6 h reaction time catalyzed by CYP154C5 F92A. The peaks at 9.7 min and 4.3 min correspond to the substrate progesterone and the product 16α-hydroxyprogesterone, respectively. The peak at 5.6 min represents a new product identified as 21-hydroxylated progesterone by NMR analysis.

21-hydroxyprogesterone (11-deoxycorticosterone) ^{1}H-NMR (500 MHz, $CDCl_3$): δ 5.76 (s, 1H), 4.30 - 4.14 (m, 2H), 3.30 (s, 1H), 2.55 - 2.18 (m, 7H), 2.06 (dt, J = 13.6, 4.2, 1H), 1.96 (dd, J = 9.3, 6.4, 1H), 1.89 (ddt, J = 11.9, 5.8, 2.7, 1H), 1.83 - 1.54 (m, 6H), 1.21 (s, 4H), 1.10 (qt, J = 12.9, 6.6, 2H), 1.00 (td, J = 11.3, 4.0, 1H), 0.91 (t, J = 6.8 Hz, 1H), 0.85 (dd, J = 13.4, 6.2, 1H), 0.72 (s, 3H). ^{13}C-NMR (126 MHz, $CDCl_3$): 210.2 (20-C), 199.5 (3-C), 170.8 (5-C), 124.0 (4-C), 69.43 (21-C), 59.1 (17-C), 56.1 (14-C), 53.6 (9-C), 44.7 (13-C), 38.6 (10-C), 38.4 (12-C), 35.7 (8-C), 35.6 (1-C), 33.9 (2-C), 32.7 (6-C), 31.9 (7-C), 24.5 (16-C), 22.9 (15-C), 20.9 (11-C), 17.3 (19-C), 13.5 (18-C).

Obtained NMR data of formed 11-deoxycorticosterone are consistent with previously published data.[1, 2]

b. Conversion of ethioallocholane (9) by CYP154C5 WT

A

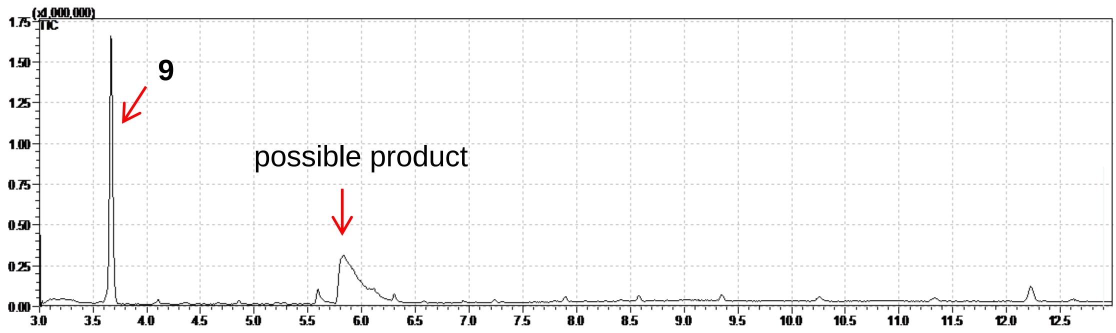

B

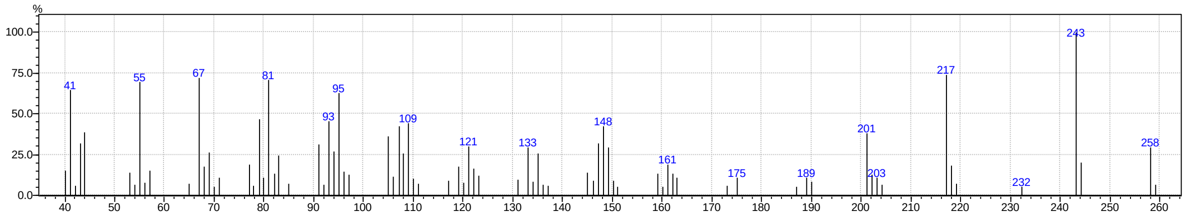

Figure S15. GC-MS result of ethioallocholane (**9**) conversion by CYP154C5. **A:** Chromatogram of reaction; **B:** MS of possible product peak at RT = 5.9 min with a maximum molecular ion peak of m/z = 258, equivalent to $[M]^+$-2. This likely corresponds to a hydroxylated product of **9** that undergoes water elimination during GC-MS measurement (which was already observed for other hydroxylated steroids previously).

c. Conversion of 3-deoxydehydroepiandrosterone (10) by CYP154C5 WT

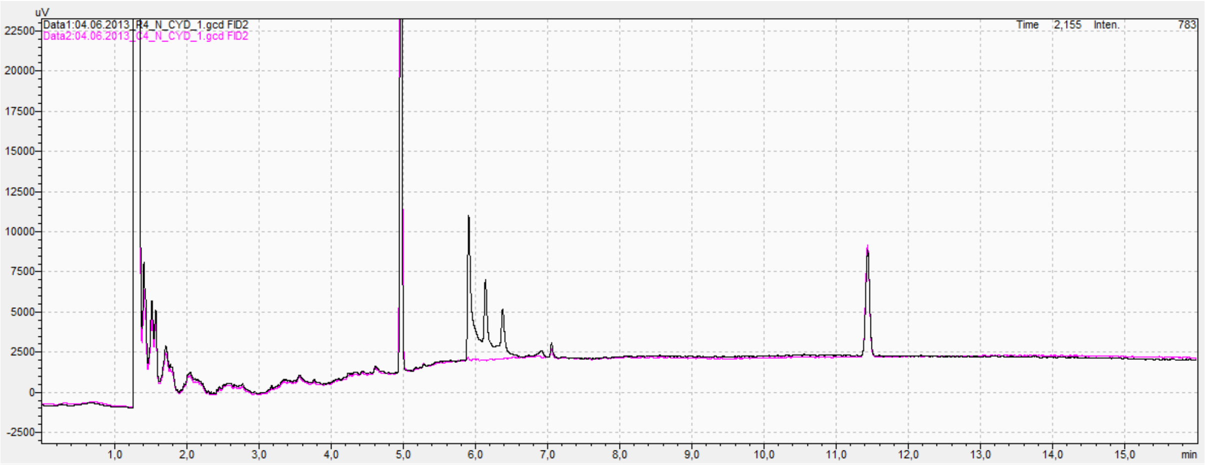

Figure S16. GC chromatogram (black) of 3-deoxydehydroepiandrosterone (**10**) conversion after 16 h catalysed by CYP154C5 wild type. For comparison, the GC chromatogram of the respective control reaction (magenta) lacking CYP154C5 is shown as well. The peak at 4.97 min corresponds to the substrate whereas the peaks at 5.8 to 6.6 min represent reaction products.

<u>Main product:</u>

16α-hydroxy-3-deoxydehydroepiandrosterone ^{1}H-NMR (600 MHz, DMSO): δ 5.34 (1H, d, *J*= 5.6, 16-OH), 5.26 (1H, d, *J*=5.1, 6-H), 4.21 (1H, dd, J_d= 8.4, J_d= 5.9, 16-βH), 2.22 (1H, m, 4α-H), 2.05-1.95 (2H, m, 4β-H and 7β-H), 1.91 (1H, dt, J_t= 13.7, J_d= 8.5, 15β-H), 1.81 (1H, d, *J*= 12.7, 1α-H), 1.73-1.51 (6H, m, 2α-H, 3β-H, 8β-H, 11α-H, 12β-H and 15α-H), 1.50-1.43 (2H, m, 2β-H and 14α-H), 1.39 (1H, dq, J_q= 13.4, J_d= 4.6, 11β-H), 1.31-1.21 (2H, m, 7α-H, 12α-H), 1.14 (1H, m, 3α-H), 1.03-0.95 (5H, m, 1β-H, 9α-H and 19-Me), 0.86 (3H, m, 18-Me). ^{13}C-NMR (150 MHz, DMSO): δ 118.9 (6-C), 70.6 (16-C), 50.6 (9-C), 48.7 (14-C), 47.2 (13-C), 39.4 (1-C), 37.6 (10-C), 32.8 (4-C), 32.1 (7-C), 31.8 (12-C), 31.3 (8-C), 30.5 (15-C), 27.9 (3-C), 22.5 (2-C), 19.8 (11-C), 19.7 (19-C), 14.3 (18-C). HRMS (EI+): *m/z* found: 288.20791 (*m/z* calculated for $C_{19}H_{28}O_2$: 288.2089).

A

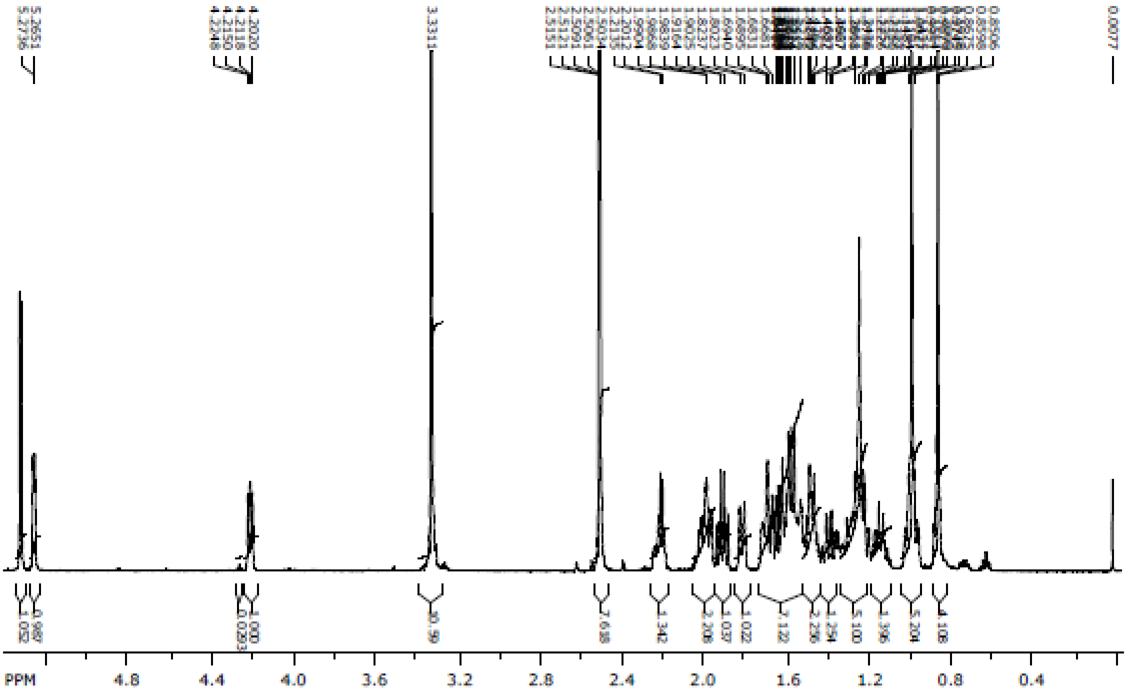
PPM
4.8
4.4
4.0
3.6
3.2
2.8
2.4
2.0
1.6
1.2
0.8
0.4

B

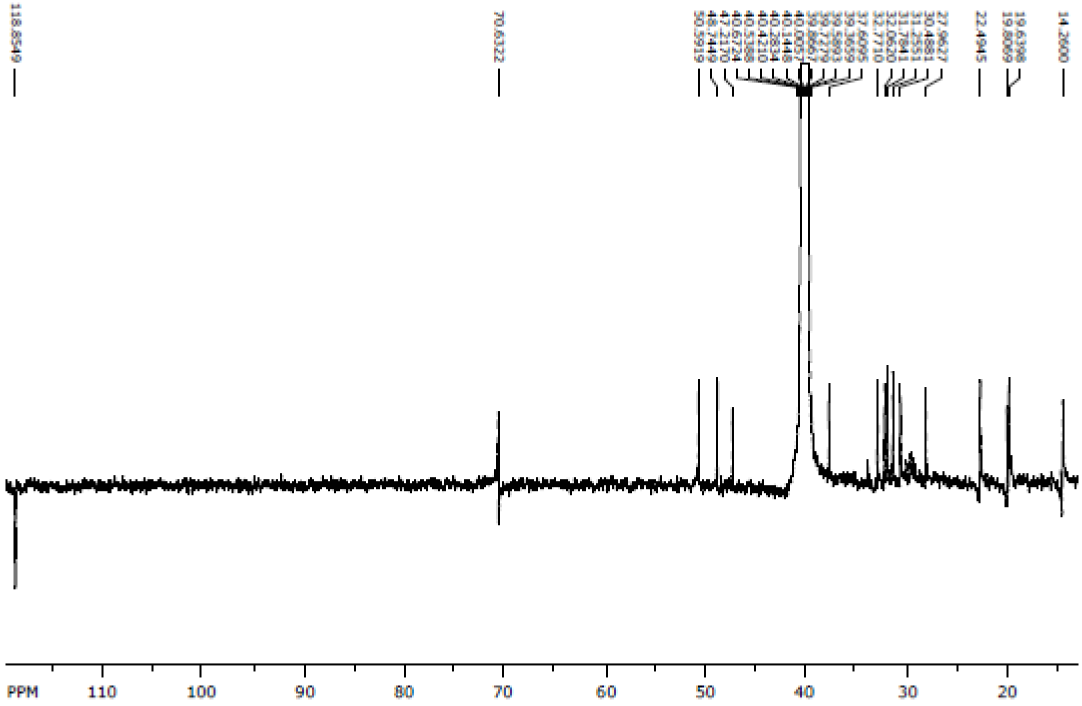
PPM
110
100
90
80
70
60
50
40
30
20

C

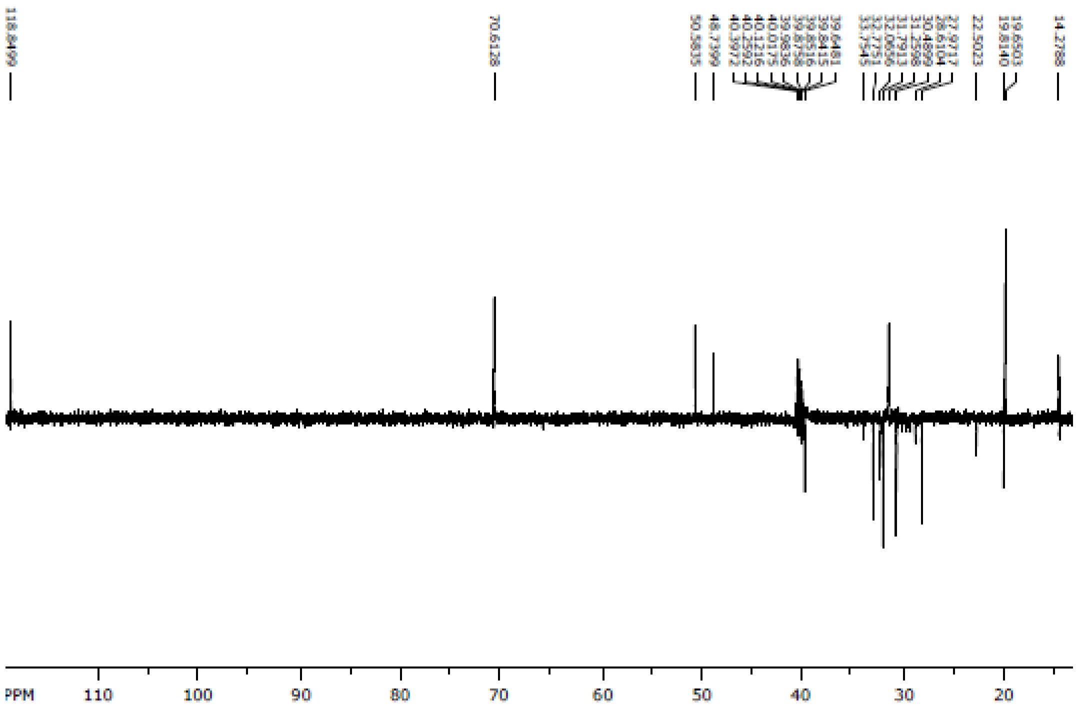
PPM
110
100
90
80
70
60
50
40
30
20

D

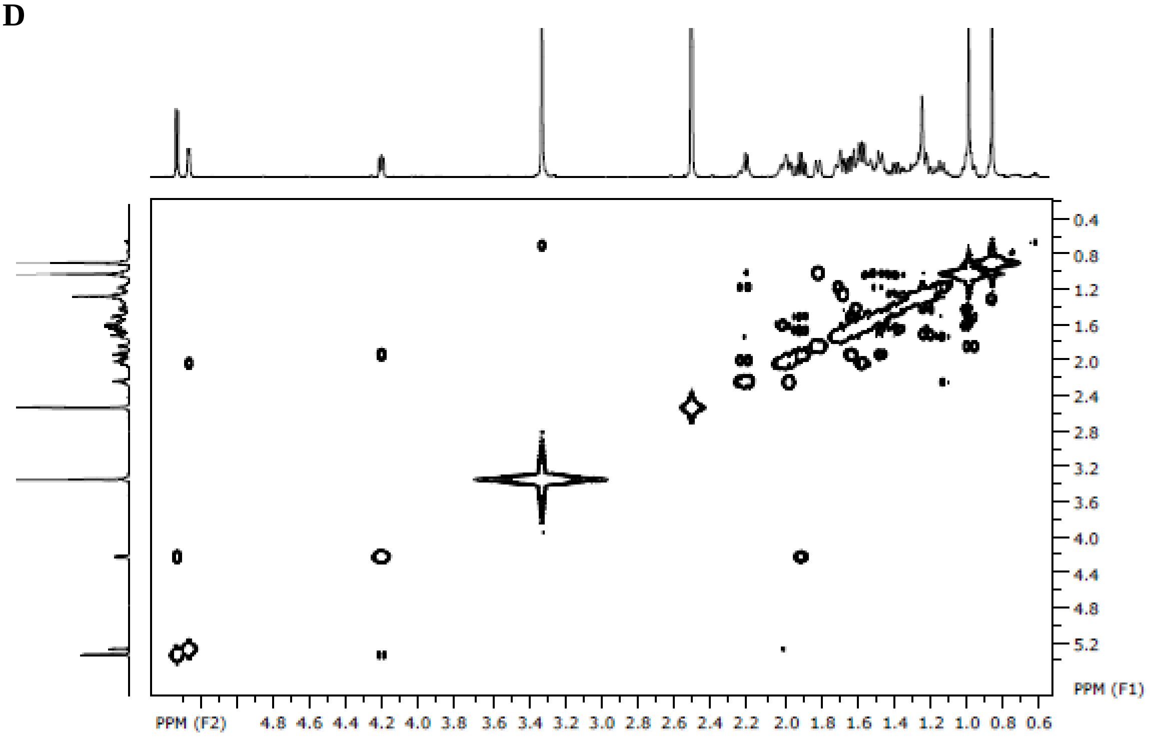
PPM (F2)
4.8 4.6 4.4 4.2 4.0 3.8 3.6 3.4 3.2 3.0 2.8 2.6 2.4 2.2 2.0 1.8 1.6 1.4 1.2 1.0 0.8 0.6
PPM (F1)
0.4 0.8 1.2 1.6 2.0 2.4 2.8 3.2 3.6 4.0 4.4 4.8 5.2

E

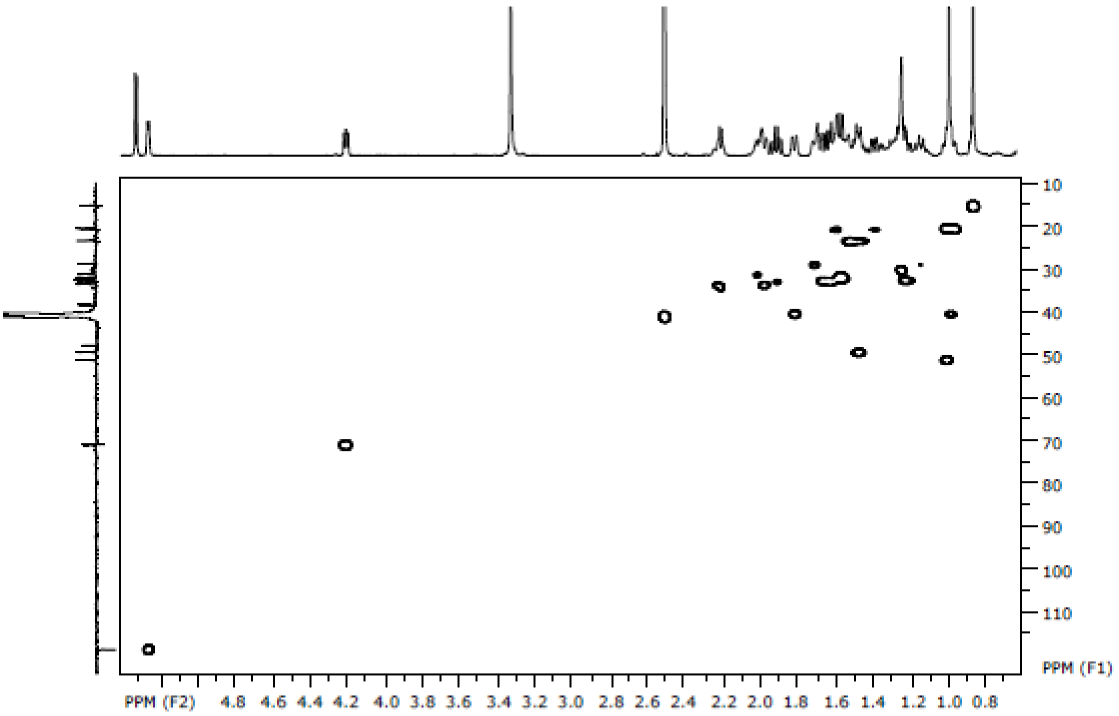

F

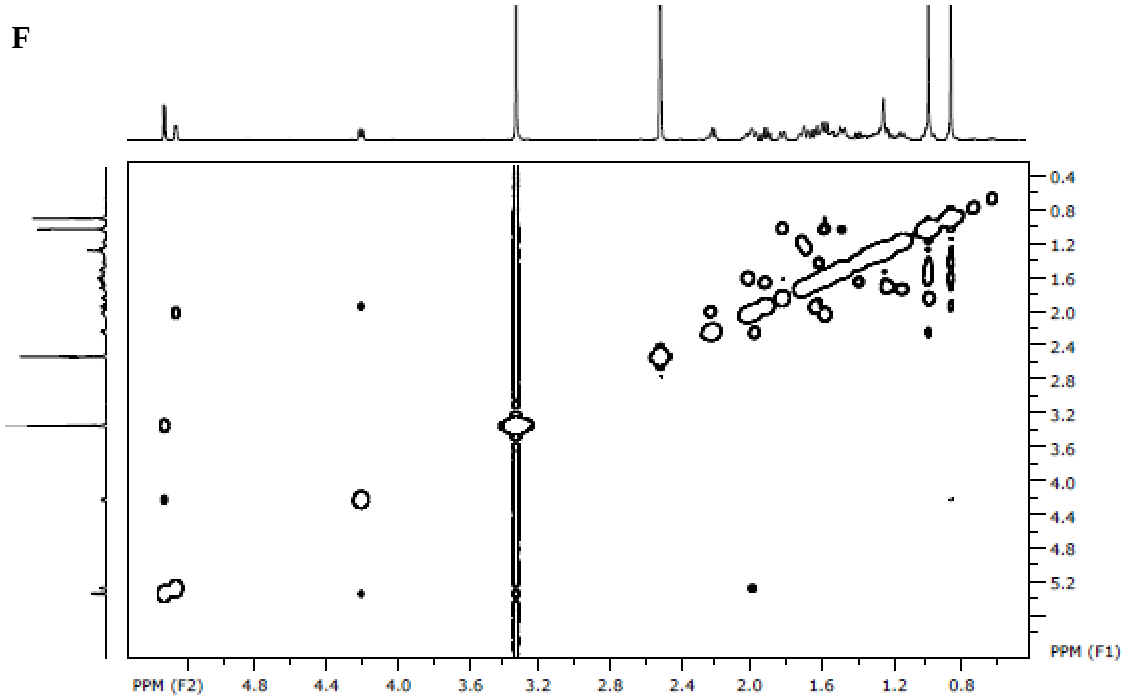

Figure S17. NMR spectra of 16α-hydroxy-3-deoxydehydroepiandrosterone; **A**: ^{1}H, **B**: ^{13}C, **C**: DEPT135, **D**: COSY, **E**: HSQC, **F**: NOESY.

Additional 3-deoxydehydroepiandrosterone product:

GC-MS analysis of the product gave a peak with RT = 8.7 min with a maximum molecular ion peak of m/z = 304, equivalent to $[M]^{+}$+32 (Figure S18). This result suggests a possible double hydroxylation of substrate **10**. This is consistent with the obtained ^{1}H-NMR data showing two different hydroxylation signals at 4.42 and 4.05 ppm (Figure S19A). Additionally, using HSQC-NMR analysis, these two proton signals were found to couple with carbon signals at 71.7 and 66.5 ppm corresponding to C̲H-OH (Figure S19B). Unfortunately, further structure elucidation was not possible due to the low amounts of purified product and, hence, a low resolution of the NMR spectra. HRMS (EI+) measurement gave *m/z* of 304.20201 (*m/z* calculated for $C_{19}H_{28}O_3$: 304.2038) consistent with two hydroxylation sites.

A

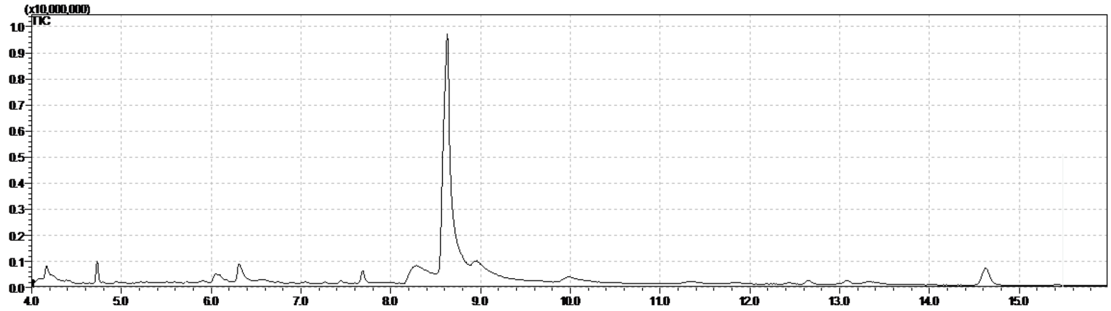

B

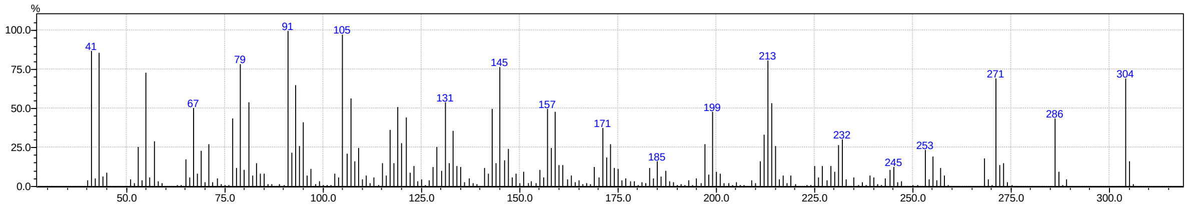

Figure S18. GC-MS result of the potential dihydroxylated product in the conversion of **10. A:** Chromatogram of the product. **B:** MS of the product peak at RT = 8.7 min.

A

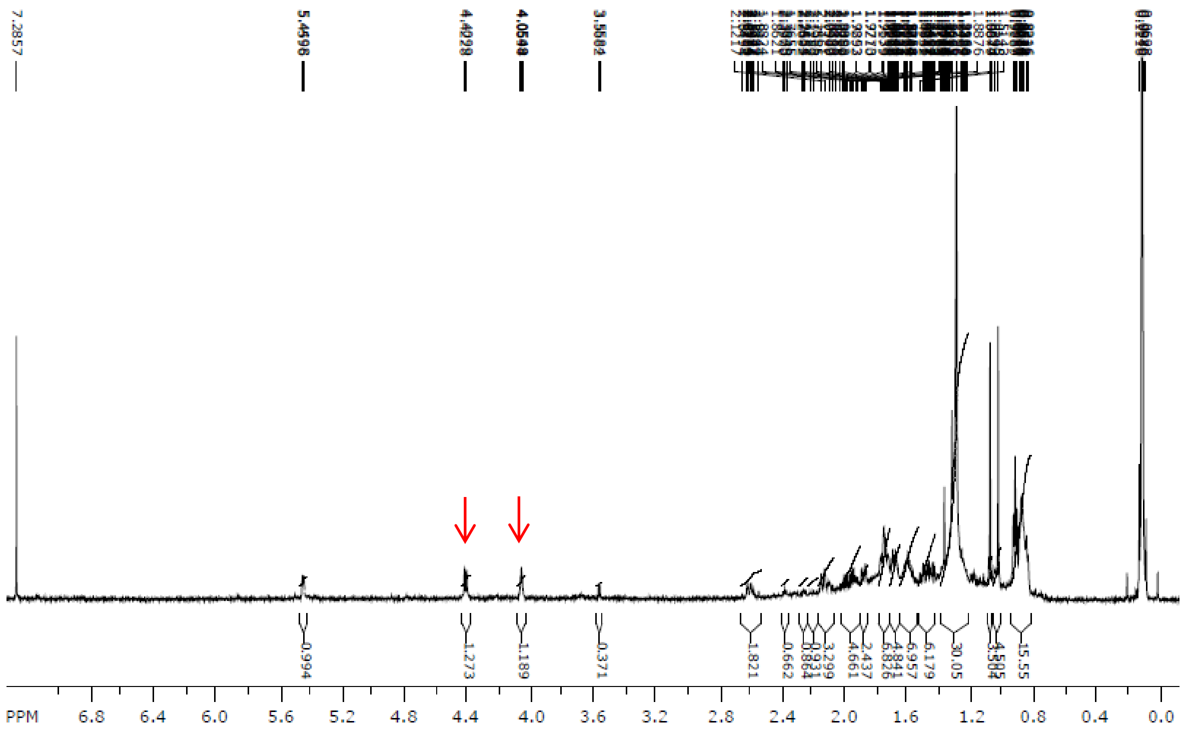

B

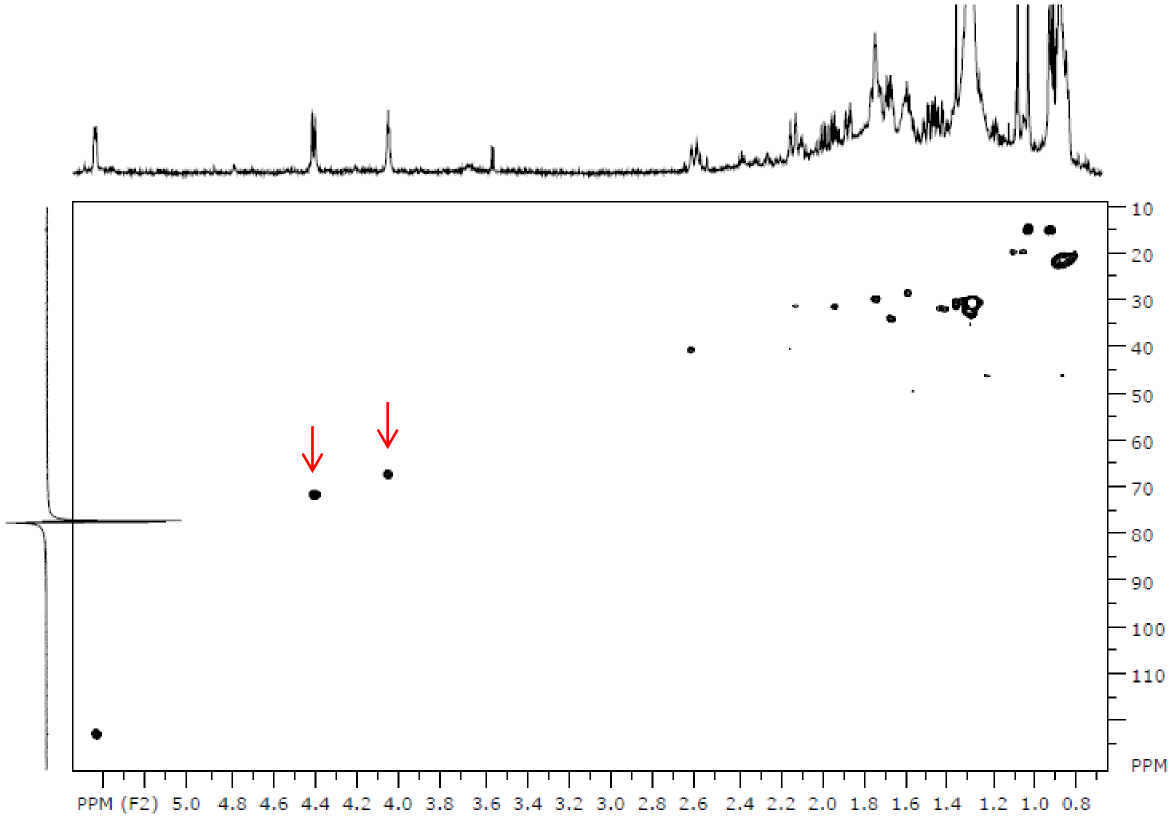

Figure S19. NMR spectra of the dihydroxy-3-deoxydehydroepiandrosterone product; **A**: ^{1}H, **B**: HSQC.

d. Conversion of 5α-androstan-3-one (11) by CYP154C5 WT

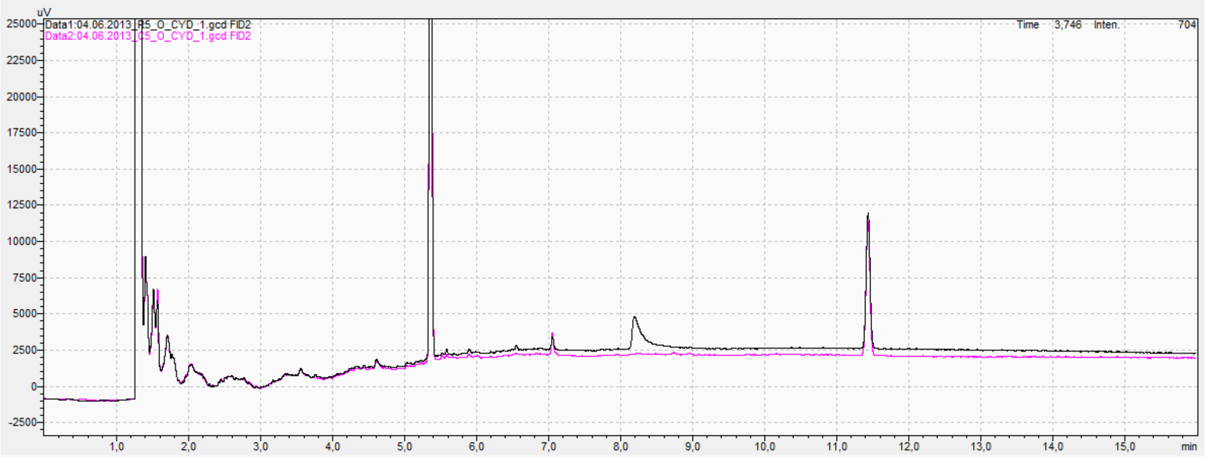

Figure S20. GC chromatogram (black) of 5α-androstan-3-one (**11**) conversion after 16 h catalysed by CYP154C5 wild type. The peak at 5.37 min corresponds to the substrate and the formed product eluted at 8.24 min. For comparison, the GC chromatogram of the respective control reaction (magenta) lacking CYP154C5 is shown as well.

Assignment of the hydroxylation product according to NMR measurements

The ^{1}H-NMR spectrum of the product shows one signal at 4.5 ppm, corresponding to the proton at a hydroxylated carbon atom. Moreover, in the respective ^{13}C-NMR spectrum, signals for C15 and C16 of the hydroxylation product are shifted significantly low-field compared to substrate **11**, indicating that the hydroxylation had occurred at one of these carbons. According to HSQC and ^{1}H-^{1}H-COSY spectra, the proton signal at 4.5 ppm was found to couple with the carbon signal at 71.6 ppm as well as with proton signals at 1.18, 1.7 and 2.1 ppm. Based on the ^{1}H-^{1}H-COSY spectrum in combination with the HSQC spectrum as well as comparison with NMR data of the substrate, these proton signals could be assigned to correspond to 14-H, one 17-H and one 16-H, respectively. Hence, the hydroxylation site was concluded to be C15. The fact that the 15-H coupled only with one 16-H is in agreement with the Karplus relation, meaning that the coupling with the other 16-H reaches zero if the dihedral angle between both protons approaches 90°. Instead, a long-range coupling with one 17-H is observed, which is well-known for 5-membered rings. In the NOESY spectrum of this compound, only a cross peak of the 15-H signal with a multiplet from 1.67-1.77 ppm is observed. This multiplet was assigned to correspond to signals of 7β-H, 12β-H and 17β-H according to HSQC, ^{1}H-^{1}H-COSY and NOESY spectra. Accordingly, the 15-H at 4.5 ppm was concluded to have β-orientation as well. This means that the hydroxyl group at C15 will have α-orientation, which is in agreement with obtained structural data of CYP154C5 in complex with steroid **11** as well as results from MD simulation.

15α-hydroxy-5α-androstan-3-one ^{1}H-NMR (600 MHz, $CDCl_3$): δ 4.50 (1H, m, 15β-H), 2.45-2.23 (2H, m, 2α-H, 2β-H), 2.29 (1H, t, J_t= 14.3, 4α-H), 2.14-2.06 (2H, m, 4β-H, 16α-H), 2.04 (1H, dq, J_q= 6.8 J_d= 2.3, 1α-H), 1.77-1.53 (6H, m, 5α-H, 7β-H, 11-H, 12β-H, 17α-H, 17β-H), 1.44-1.25 (7H, m, 1β-H, 6α-H, 6β-H, 8β-H, 11-H, 12α-H, 16β-H), 1.18 (1H, dd, J_d= 12.5 J_d= 5.9, 14α-H), 1.06-0.98 (4H, m, 7α-H, 19-CH_3), 0.85 (1H, m, 9α-H), 0.75 (3H, s, 18-CH_3). ^{13}C-NMR (150 MHz, $CDCl_3$): δ 211.2 (3-C), 71.7 (15-C), 54.0 (9-C), 52 (14-C), 52 (16-C), 46.6 (5-C), 44.7 (4-C), 41.9 (13-C), 38.6 (1-C), 38.5 (2-C), 38.2 (12-C), 37.3 (17-C), 35.8 (10-C), 35.2 (8-C), 31.9 (7-C), 28.9 (6-C), 21.1 (11-

C), 18.7 (19-C), 11.5 (18-C). HRMS (EI+): *m/z* found: 290.22346 (*m/z* calculated for $C_{19}H_{30}O_2$: 288.2246).

A

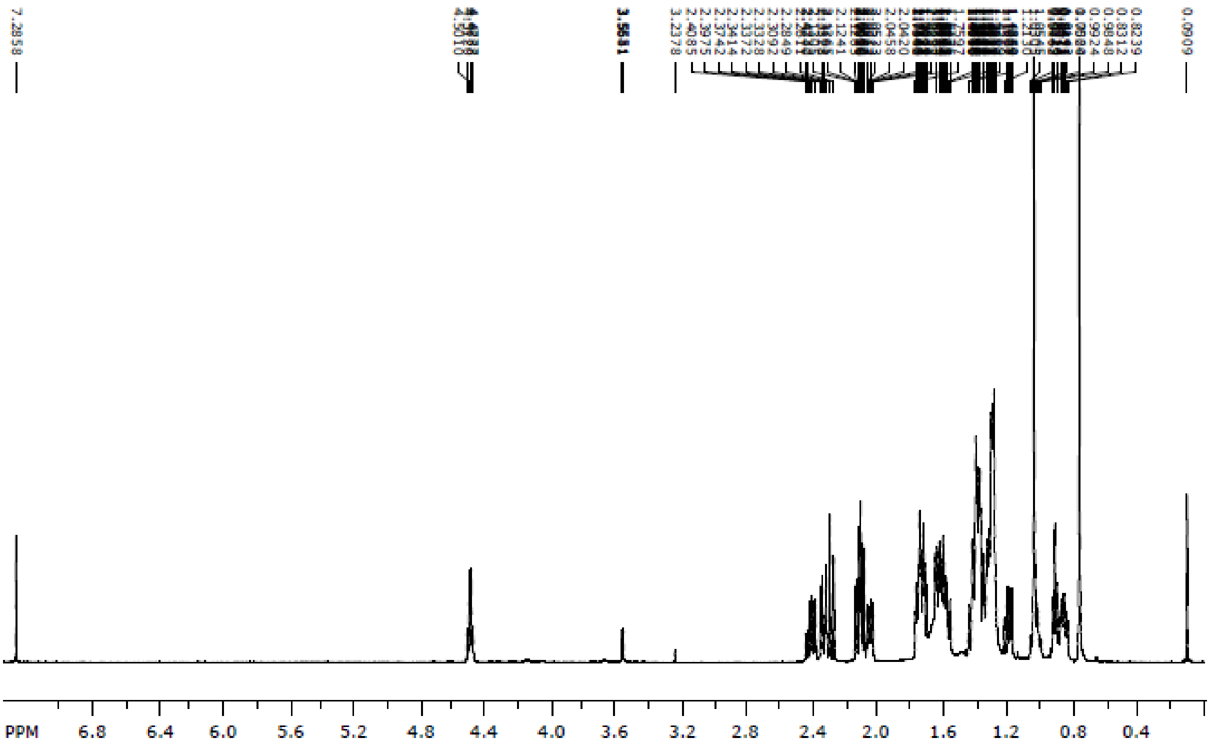

B

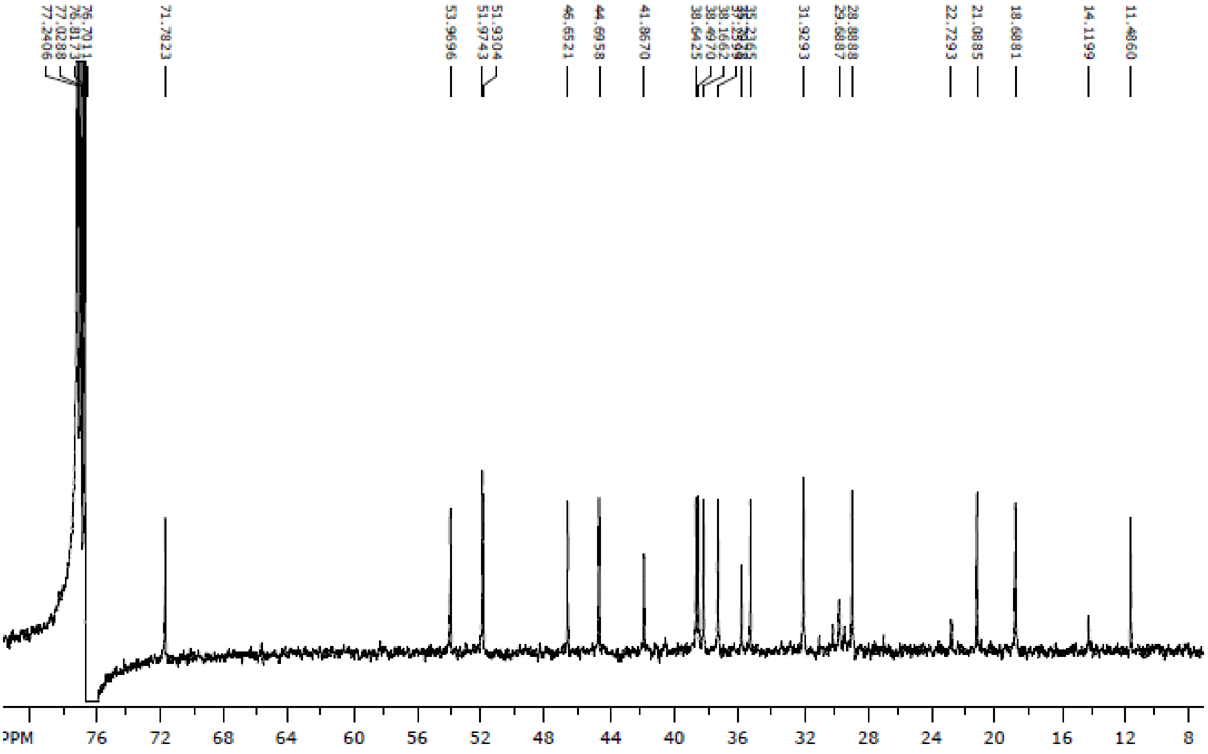

C

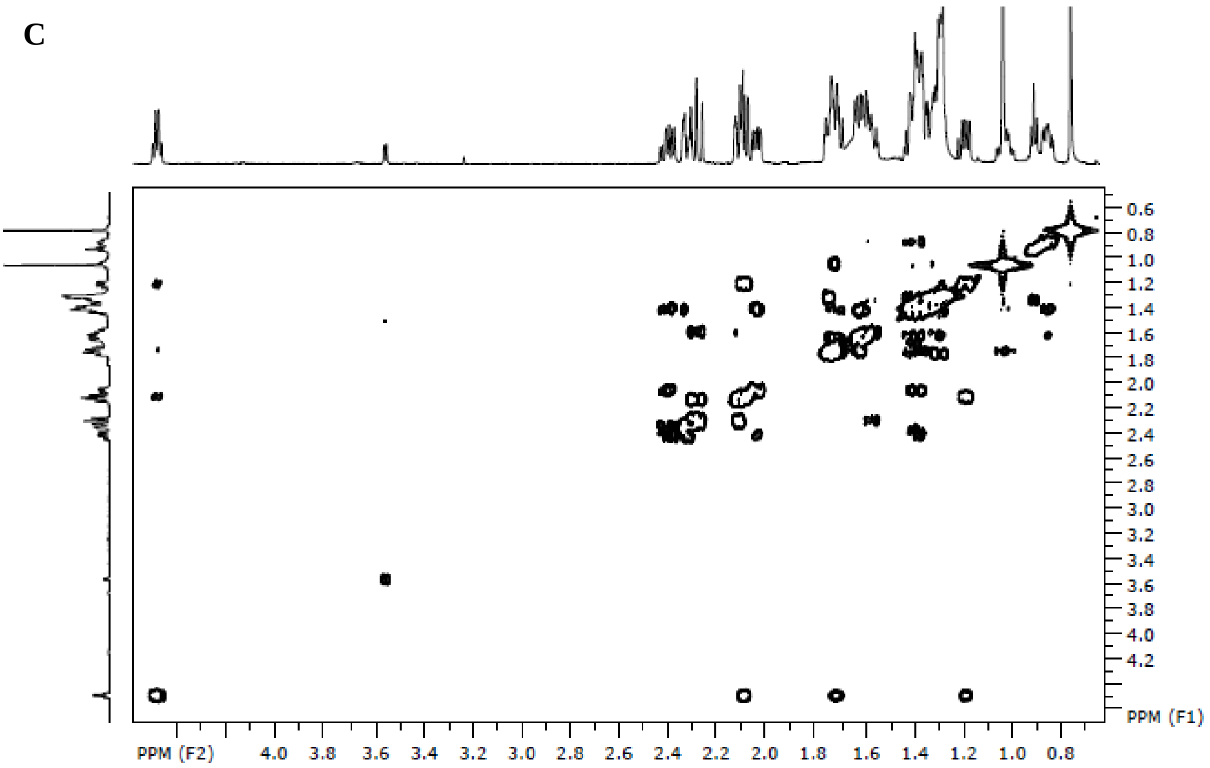
PPM (F2)
4.0 3.8 3.6 3.4 3.2 3.0 2.8 2.6 2.4 2.2 2.0 1.8 1.6 1.4 1.2 1.0 0.8
PPM (F1)
0.6 0.8 1.0 1.2 1.4 1.6 1.8 2.0 2.2 2.4 2.6 2.8 3.0 3.2 3.4 3.6 3.8 4.0 4.2

D

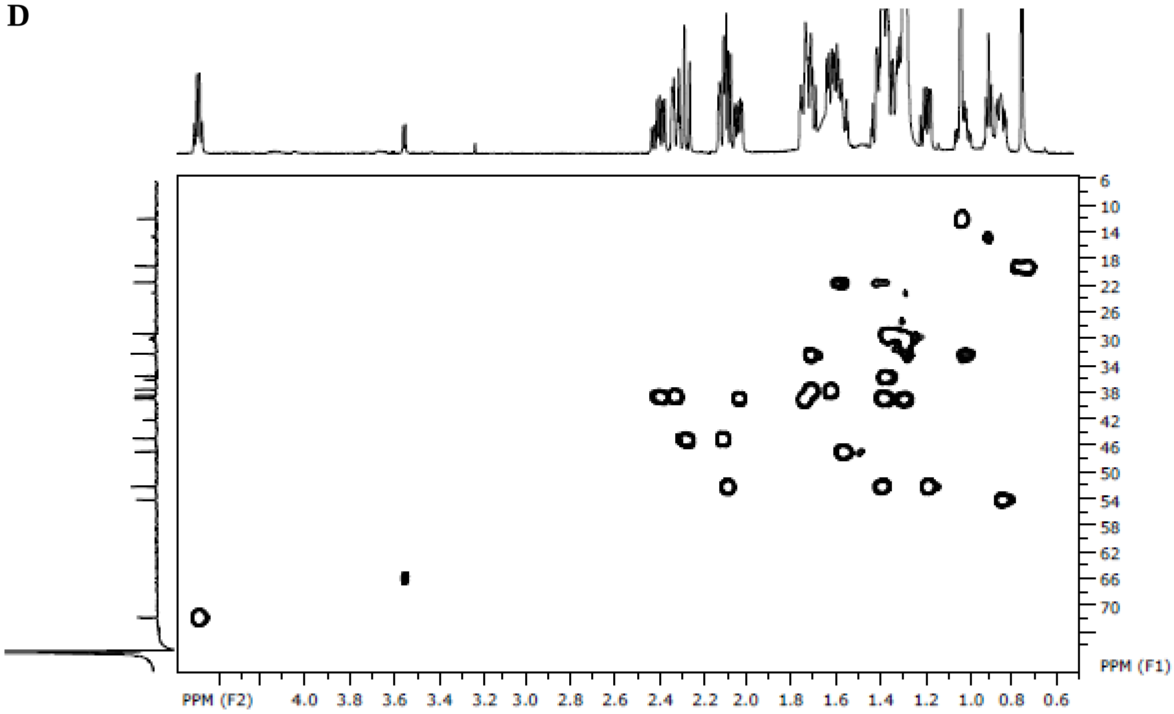
PPM (F2)
4.0 3.8 3.6 3.4 3.2 3.0 2.8 2.6 2.4 2.2 2.0 1.8 1.6 1.4 1.2 1.0 0.8 0.6
PPM (F1)
6 10 14 18 22 26 30 34 38 42 46 50 54 58 62 66 70

E

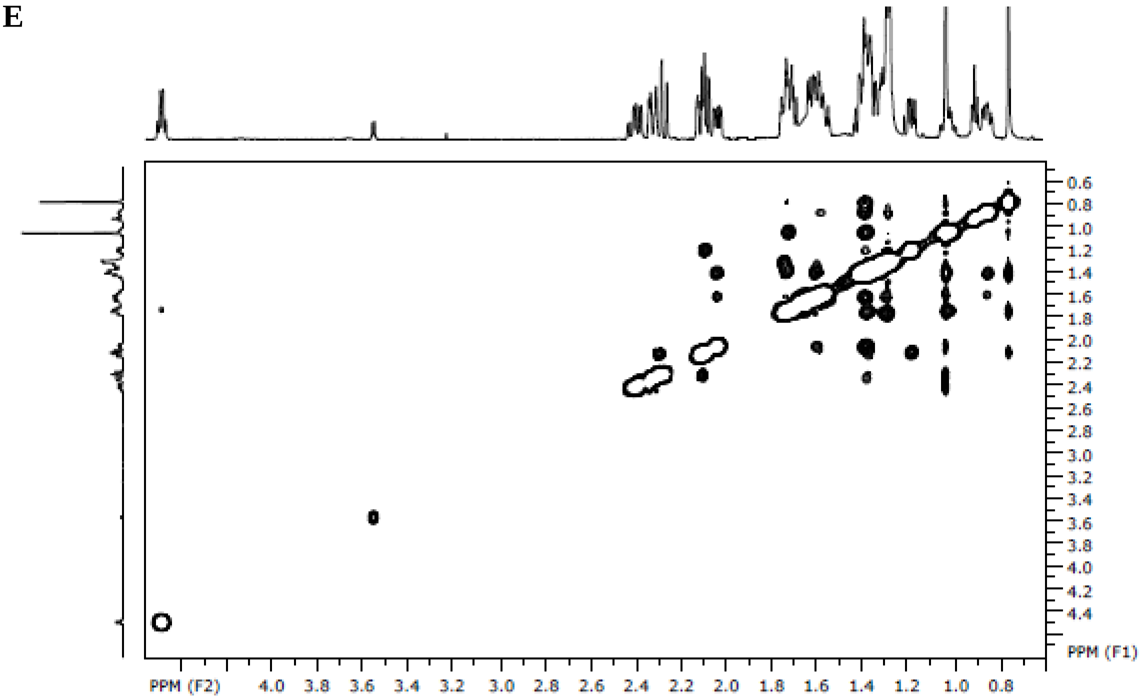

Figure S21. NMR spectra of 15α-hydroxy-5α-androstan-3-one; **A**: ^{1}H, **B**: ^{13}C, **C**: COSY, **D**: HSQC, **E**: NOESY.

References

[1] R.M. de Pádua, N. Meitinger, F.J.D. de Souza Filho, R. Waibel, P. Gmeiner, F. Castro Braga, W. Kreis, *Steroids* **2012**, *77*, 1373-1380.

[2] M.F. Grostic, K.L. Rinehart Jr., *J. Org. Chem.* **1968**, *33*, 1740-1746.
